# Tactile Contact as a Marketing Tool for Improving an HIV/STD Education Program’s Compliance / Retention with Crack Cocaine Users

**Published:** 2020-01-20

**Authors:** Ralph Jay Johnson

**Affiliations:** University of Texas MD, Anderson Cancer Center; Chaplain Texas Medical Center Catholic Chaplaincy Corp, Texas

**Keywords:** tactile contact, tactile stimulation, touching, program compliance, anchoring, marketing tool

## Abstract

**Background:**

This research brief reports results from an exploratory pilot study on the use of socially acceptable touch in a public setting that accompanies a request to improve program compliance with “street level” crack cocaine users.

**Methods:**

Study participants consisted of 120 crack cocaine-using participants in a larger community-based HIV/STD prevention and research program targeting at-risk African-Americans. They were required to return for a series of four booster health education sessions over 2–5 days and 6 month and 1 year follow-up assessments. The most difficult aspect of this program was no-shows for the second booster session; study participants who attended at least two sessions were much more likely to attend all sessions and complete the entire lengthy program. The program director randomly approached some participants after the first visit in a public setting and briefly touched them as part of a handshake; then, the director asked them to return for their follow-up sessions. Whether they were approached or not was random. Analysis comprised descriptive and non-parametric statistics.

**Results:**

Ninety-three percent of participants who were asked to return and were touched returned for the second session; only 75% returned who had been asked to do so but were not touched. A statistically significant difference favored being touched and complying, as measured by second-session returning participants (p < .01), though it appeared the touch / request had more of a preventive than a promotional effect. Extraneous demographic and background factors were ruled out with the exception of age (older participants), which contributed slightly.

**Conclusions:**

Results suggest that a request “anchored” to a socially acceptable public touch is promising in terms of improving program participation and engagement. Limitations and implications for future research are discussed.

## Introduction

Keeping participants continuously engaged in programs can be a difficult aspect of treatment services at best.[1–7] Nevertheless; common sense dictates; and research has shown; that the longer participants actively participate in programs; the better their prognosis.[8–15] A challenging task is communicating to participants the importance of sticking with a program.[13; 16–19] One of the most primal modes of communication is touch: research shows that; along with a suggestion or request; touch has a synergistic effect on compliance.+

In a series of foundational experiments in a variety of natural settings (e.g.; medication non-compliance; product sales; solicitations for gifts or favors; or help; information; and even free rides and violations of prohibited behavior; etc.) over several years; Gueguen and associates[20–25] convincingly demonstrated that a brief touch and a direct gaze accompanied by a request had a positive influence on compliance—despite the size of the request from the person doing the touching and whether recipients were even aware they were touched at all. Indeed; Hornik;[26] Smith; Gier; and Willis;[27] Willis and Hamm;[28] and Crusco and Wetzel [29] found that tactile contact enhanced spontaneous compliance and improved sales—even when no request was made.

Nevertheless; though a logical next step; few; if any; researchers have evaluated the practical and cost-effective use of touch accompanied by a direct gaze in requests to proactively encourage continued attendance of intervention programs in the interest of boosting retention—that is; as a program marketing tool. This is unusual given the number of marketing articles urging the “personal touch” to improve program attendance and compliance and customer/brand loyalty in general.[30–37] To test this effect; a pilot sub-study within a larger demonstration and research program was carried out. Participants in a community-based HIV/STD prevention project—who were currently using crack cocaine—were asked to return to a storefront outreach center after their initial visits for a series of four sequential daily “booster” sessions of health education over 4–5 days. Upon leaving the center; some participants were approached by the project/clinic director; who briefly touched them with a “supported” congratulatory handshake and; looking them directly in the eyes; asked that they return for their next scheduled visit.

The expectation was that those who were touched in this manner were more likely to comply than those who were not. Though the research question may not have been novel; its next logical extension—specifically; practical application and programmatic evaluation— was unique and innovative; and constitute an advancement. The purpose of this research article is to briefly report the results of the exploratory pilot sub-study.

## Methods

### Study Volunteers

Seventy-five male and 45 female (N = 120)++ African American program participants served as study volunteers.

### Participants

Study participants were part of a larger research project that compared different modes of health education to improve condom use and safe-sex practices among African Americans currently smoking crack cocaine and engaging in risky sex. Volunteers had been recruited to a storefront outreach center where they were given a baseline interview and an initial health education session. They were expected to attend four more daily “booster” sessions on four subsequent days. Then they were supposed to return for reevaluation six and 12 months later.

However; some simply did not return after the initial session despite remuneration incentives for each attendance and repeated attempts to re-contact and encourage them to return. The daunting challenge was motivating participants; who were substantially unreliable; transient; and itinerant; to return the next day for their second session.^**cf.2**^ Excluded from this marketing pilot study were those who failed to return because of a verified and documented extraneous event or factor (e.g.; arrest and long-term incarceration; permanent relocation; hiding and on the run; death; or extended in-patient drug treatment).

Table I provides a demographic and Social-Economic-Status (SES) profile of the study participants in order to assess whether there were significant differences between the Touched and Not Touched groups that could influence subsequent return rates. Generally; the two groups were homogenous. Nevertheless; there were slight differences between them. It should be noted that selection into either group was random and not based on these criteria. Thus; there was no way to know how the two groups fared regarding these criteria; or even their return rates; until nearing the study’s end. In this sense; the study was “blinded.”

Also; given how study participants were included; it would have been nearly impossible to achieve a perfect match; although the similarity of the two groups was remarkable. Table I reveals that the differences in terms of demographics and SES were slight; if not inconsequential. More of the Touched group were females (4%); lived in permanent housing (1%); and were older; but were slightly less educated and with less income (2%). Whereas; the Not Touched group somewhat represented a very mild anti-thesis: more Male (2%); more living in transitory housing (2%); they were younger; and they had slightly less education; but had more income (2%). Remarkably; for street level crack cocaine users; many program participants had at least a GED (high-school equivalency exam certificate); if not a high school diploma and some college (usually a couple of years of community college).

### Procedure

As part of normal office protocol; the “script” consisted of; after participants’ initial session and when they were leaving the center (see Digital Picture); of the following:

(1)The project/clinic director would catch them at or outside the office door; (2) introduce himself; (3) extend a hand to offer a congratulatory handshake on “completing the first session”; (4) if the person extended his or her hand back and shook the director’s hand; (5) the director would use his other hand to steady the handshake by touching the person’s hand/forearm; (6) then the director would look directly into the person’s eyes and (7) request that the person come back for the next session. (Naturally; the project/clinic director did practice hand hygiene between encounters to reduce the risk of hand-borne infectious disease transmission.)

### Digital Picture of Intervention

**Note:** Though visual facial features in the photo were redacted or concealed; informed consent included permission for visual recording followed/supported by verbal assent for both parties.


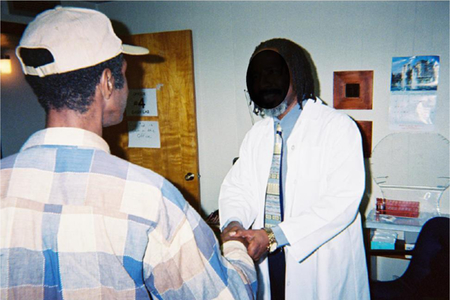


An attractive feature of this procedure as a program marketing tool is that it involved ordinary; polite public behavior that did not violate social norms; was not offensive; and occurred with participants’ permission. The hope was that this would help improve return rates for the second session; and ideally; once the participants got into the habit of returning; overall retention rates for the three remaining sessions would improve; as would the subsequent 6- and 12-month return rates for re-evaluation visits. One of the measures of the program’s success was Continuity of Care. The assumption was that; if the program could encourage this rather difficult; intransient; and intractable target population to return multiple times to attend “booster” follow-up health education and risk reduction sessions; then that increased the possibility they would retain the instructional material over time.^**cf**.^[7;9;10;12]

Nevertheless; the project director could not reach everyone. By default; some participants met the director and received the touching and request while others did not; which was documented. Whether or not a client received the touching and request was random. Prior to their program enrollment; informed consent was obtained from all participants; and those who were touched were given the opportunity to debrief regarding this particular study. Only three requested the debriefing.

The study was reviewed and approved by the University of Texas-School of Public Health Human Subjects Protections Committee and adequate informed consent to participate was obtained from study participants.

#### Data Collection and Analysis:

Assignment to the Touched and Not Touched groups was noted in the study participants’ program files; which also included their progress through the program’s sessions. Study participants were recruited until 60 was reached for each group. Data were entered electronically and analysis consisted of using descriptive statistics and a Chi-Square and Odds Ratio. In accordance with the nominal outcome measure; binomial multiple logistic regression was employed as a postscript to examine possible explanatory influence of extraneous co-factors.

## Results

Analysis showed that 93% (56 of 60) of the participants who had been touched returned for a second session as opposed to 75% (45 of 60) who had not been touched. The most dramatic results were among those who did not return: 80% (15 out of 19) had not been touched while 20% (4 out of 19) had been touched.

The difference between the rates was significant [*X*_1_^2^ (N = 120) = 7.567; *p* <.01]. There were no significant differences between demographic and background variables except for age. Seventy percent of the older participants (older than 30) returned while only about 15% of the younger ones (18–30 years old) returned [*X*_1_^2^ (N = 120) = 7.22; *p* <.01]. Logistic regression revealed that touching (*B* = 1.557; *p* = .01) appeared to positively affect rates of returning for the second session; and age (*B* = .116; *p* < .03) only contributed slightly though its effect could not be ruled out. Overall; 90 participants who attended their second session also attended their subsequent three sessions and their 6- (90%) and 81 of those (81%) their re-evaluations. So; theoretically a touched participant would have a 5 times greater chance of returning for their second session and completing the entire intervention.

## Conclusions

The study’s results support the contentions of previous researchers^**25–29**^; but extended them through practical application. Specifically; socially acceptable public touch had a significant effect on program compliance / retention when anchored to a request for the participants; defined here as coming back for a return visit. The most dramatic difference was between those who were not touched and failed to return (21% vs. 79%) as opposed to those who were not touched and returned (45% vs. 50%). Apparently; the touching had a preventive rather than a promoting effect. Nevertheless; these results support a practical; efficient; and cost-effective application of combining socially acceptable touching with a request or instruction to boost compliance for a population known to be particularly difficult in terms of program participation and compliance.[38–40] The potential adaptations of this simple technique are unlimited for improving retention in programs that involve participants’ repeated involvement over time. The technique is not harmful or impolite and allows for a demonstration of genuine interest and even appreciation toward participants’ continued engagement with a program.

## Limitations

The findings were not without limitations. First; the project/clinic director was male. Future research should examine whether gender or a status makes a significant difference in the return rate. Second; little is known about the recipients’ perceptions of the touching.^cf.25^ Further investigations should consider whether and how (1) the effects of touch are mediated through the recipients’ positive perceptions; (2) the awareness of being touched makes a difference; and (3) what being touched means to recipients.

Third; compliance was measured only in terms of returning for the second visit. Further inquiry might look into whether touch and a request improve compliance with other types of requests; other populations; and other program settings. Fourth; the population represented one ethnicity in one city; and one programmatic target population. Future research should investigate cultural differences in terms of the social appropriateness and influence of touching accompanied by a request in different settings.

Fifth; the relevance of age should be appraised more carefully; as should possible other unknown extraneous mediating factors. Sixth; the confidence intervals for the Odd Ratio; though statistically significant; were relatively substantial. This is probably due to the study being low statistically powered (but not underpowered)—hence further; larger; and more inclusive studies are suggested. This also would accommodate expanded analysis.

Finally; critics might argue that this study was merely reaffirmation of desirability bias at work in research. That is; the participants were doing nothing more than responding to research cues (e.g.; the Hawthorne effect+++ revisited). However; the original intent of the study was to pilot test a socially acceptable and easy-to-use cue for managers and researchers to use to improve program compliance and retention; with less regard for the underlying mechanisms that make the behavior effective.

Despite its shortcomings; the study strongly supports the positive and promising effects of the practical deployment of benign touch in a brief public encounter on improving compliance with a request—even a relatively substantial request—in a program or clinic setting. Additionally; compliance with such an initial request has the possibility of increasing further compliance. Hopefully; limitations in light of the dated information in the literature; the results reported herein will stimulate new research to foster and refine what is known about uses of tactile contact and compliance.

+Hypnotherapists have long understood the “anchoring” power of touch accompanied by suggestion in that touch focuses attention to the area touched; thereby altering the state of consciousness and making one more open to suggestion.[15;18] In their common parlance; hypnotherapists refer to this procedure as “dropping an anchor” on someone. Note: No study participants were hypnotized; that was not the aim of the study. (https://www.mindtosucceed.com/self-hypnosis-techniques.html https://www.braindirector.com/how-to-use-anchors-in-hypnosis-and-nlp-for-building-confidence/)

++ A statistical power test revealed that the sample size provided 95% power to detect statistically significant differences at a < .05 alpha acceptance level.

+++ Two researchers studied working conditions at the Hawthorne Western Electric Works in Chicago to discover what conditions would improve employee satisfaction and productivity. They found that just about every adjustment; good or bad; improved satisfaction and productivity—until they realized that the improvements were more a function of the experiments themselves and the presence of the experimenters.[41;42]

## Figures and Tables

**Table I:** Tactile Study Participants’ Demographic and SES Profile

			N = 120						
Gender			Not Touched			Touched		Total	
	**Male**	39	32%		36	30%		75	62%
	**Female**	21	18%		24	20%		45	38%
**Age (years old)** *			Mean = 33 Range = 18–40 Std = 6			Mean = 34 Range = 19–40 Std = 6			Mean = 34 Range = 18–40 Std = 6
**Monthly Income** **									
	**$600>**		20	16%	17	14%		37	30%
	**$100–$500**		30	25%	33	27%		63	52%
	**$0–$100**		8	7%	9	8%		17	15%
	**Missing**		2	2%	1	1%		3	3%
**Years in Schoo**l			Mean = 13 Range = 7–16 Std = 2			Mean = 12 Range = 7–18 Std = 2			Mean = 12 Range = 7–18 Std = 2
**Housing**									
	**Permanent** ***	11	11%		15	12%		28	23%
	**Transitory** ****	37	37%		42	35%		87	72%
	**Streets**	1	1%		1	1%		2	2%
	**Missing**	1	1%		2	2%		3	3%

* The max upper age for entrance into the main study was 40 years old.

** Surprisingly; for street level drug users; 34% received income from some kind of full or part-time job; though the exact nature of their work was unknown. Fifty-four percent received income for “alternative” sources; including: unemployment benefits; VA/SSI disability; public assistance; spouse/sex partner; family; friends; trading sex for money; illegal sources; and “odd jobs.”

*** Permanent Housing included a house or apartment for which the Study Participant was responsible.

**** Transitory Housing was someone else’s house/apartment; hotels; rooming/boarding houses; dormitories; half-way houses; jail; or a shelter. “Streets” included homeless on the streets and vacant buildings.

**Table II:** Chi-Square/Odds Ratio Second-Session Return for Touched vs. Not Touched

		Not Touched	Touched	
Return for Session 2	No	15 (13%)	4 (3%)	19 (16%)
Return for Session 2	Yes	45 (37%)	56 (47%)	101 (84%)
		60 (50%)	60 (50%)	120 (100%)
Odd Ratio = 4.667				
CI = 1.447 – 15.046				
